# The multiple meanings of "wheezing": a questionnaire survey in Portuguese for parents and health professionals

**DOI:** 10.1186/1471-2431-11-112

**Published:** 2011-12-12

**Authors:** Ricardo M Fernandes, Brígida Robalo, Cláudia Calado, Susana Medeiros, Ana Saianda, Joana Figueira, Rui Rodrigues, Cristina Bastardo, Teresa Bandeira

**Affiliations:** 1 Department of Pediatrics, Hospital de Santa Maria, Centro Hospitalar Lisboa Norte EPE, Lisboa, Portugal; 2 Department of Pediatrics, Hospital de Faro, Faro, Portugal; 3 Joaquim Paulino Primary Care Center, Rio de Mouro, Portugal; 4 Seixal Primary Care Center, Seixal, Portugal; 5 Núcleo de Estudos da Função Respiratória, Sono e Ventilação, Hospital de Santa Maria, Centro Hospitalar Lisboa Norte EPE, Lisboa, Portugal

## Abstract

**Background:**

Most epidemiological studies on pediatric asthma rely on the report of "wheezing" in questionnaires. Our aim was to investigate the understanding of this term by parents and health professionals.

**Methods:**

A cross-sectional survey was carried out in hospital and community settings within the south of Portugal. Parents or caregivers self-completed a written questionnaire with information on social characteristics and respiratory history. Multiple choice questions assessed their understanding of "wheezing". Health professionals (physicians, nurses and physiotherapists) were given an adapted version. We used bivariate analysis and multivariate models to study associations between definitions of "wheezing" and participants' characteristics.

**Results:**

Questionnaires from 425 parents and 299 health professionals were included. The term "wheezing" was not recognized by 34% of parents, more frequently those who were younger (OR 0.4 per 10-year increment, 95% CI 0.3-0.7), had lower education (OR 3.3, 95% CI 1.5-7.4), and whose children had no history of respiratory disease (OR 4.6, 95% CI 2.5-8.7) (all ORs adjusted). 31% of parents familiar with "wheezing" either did not identify it as a sound, or did not locate it to the chest, while tactile (40%) and visual (34%) cues to identify "wheezing" were frequently used. Nurses reported using visual stimuli and overall assessments more often than physicians (p < 0.01). The geographical location was independently associated with how parents recognized and described "wheezing".

**Conclusions:**

Different meanings for "wheezing" are recognized in Portuguese language and may be influenced by education, respiratory history and regional terminology. These findings are likely applicable to other non-English languages, and suggest the need for more accurate questionnaires and additional objective measurement instruments to study the epidemiology of wheezing disorders.

## Background

Epidemiological and intervention studies in pediatric pulmonary diseases often rely on proxy's reports of respiratory symptoms in questionnaires, e.g. "wheezing". This is key to estimating asthma prevalence in large-scale studies like the International Study of Asthma and Allergies in Childhood (ISAAC), or when assessing outcomes in cohort studies or clinical trials [1-4]. Lung function testing and biomarkers are being increasingly studied to identify and describe asthma and wheezing phenotypes, but significant challenges to their widespread use remain, particularly in infants and preschoolers [5,6]. Worldwide differences and trends in prevalence estimates obtained through surveys of symptoms have major public health implications, and continue to foster debate on the risk factors and prognosis of asthma [2,7,8].

The operational definition of "wheezing" in epidemiological studies and clinical practice is usually a whistling-like sound arising from the chest [9-14]. Evidence suggests that parents and health care professionals differ in their perceptions of "wheezing" [10]. Some parents confuse "wheezing" with other respiratory sounds, while others perceive it as something other than a sound [15]. It has recently been shown that these different concepts may be a source of bias in survey results, with a considerable impact on wheezing prevalence estimates [16].

While evidence has accumulated from studies performed in English-speaking countries, little is known about this issue in other languages. Validation studies of national or international questionnaires have rarely assessed the impact of differing terminology [17,18]. However, the linguistic properties of the term "wheezing" are likely to be relevant, and these may differ among languages and cultures [19].

We conducted a survey on the meaning of a Portuguese term for "wheezing" that is used on the Portuguese version of the ISAAC - "pieira". Our primary objective was to detect whether parents and caregivers knew this term, and how they defined it. We also sought to identify parents' and children's characteristics associated with their definitions, and to compare parental perceptions with perspectives from health care professionals.

## Methods

### Study Design and Participants

We performed a cross-sectional questionnaire study in five middle- to large-sized hospitals and two primary care clinics in the Lisbon area and south of Portugal, from May 2006 to January 2007. Our aim was to recruit participants from a range of clinical settings and geographical locations. The study followed local Ethics Committees guidance and approval (Lisboa, Hospital Santa Maria).

We included a convenience sample of unselected parents or caregivers accompanying children or adolescents (< 14 years). In community settings, parents were approached when presenting to well child visits. Hospital participants were approached either before general pediatrics or pulmonology/ allergology visits, or during emergency department stay or hospital admission, irrespective of their condition. A second group of participants were health care professionals involved in pediatric care in those centers, including physicians, nurses and physiotherapists.

### Questionnaire

Participants self-completed a multi-item questionnaire written in Portuguese. The instrument included multiple-choice questions on the various domains of understanding of the term "wheezing" ("pieira") (original questionnaires available as additional files 1, 2 and 3).

Participants were asked whether they knew the term "wheezing", and if so, how they identified it (i.e. auditory, visual, tactile cues, or a sense of being unwell), where they localized it (i.e. nose, mouth, chest), and whether their child had ever had wheezing. Additionally, they were asked to identify synonyms of wheezing among other respiratory symptoms and sounds (e.g shortness of breath, snoring, ruttles). There were minor differences in the terminology used in questionnaires for parents and health care professionals. In the version for parents and caregivers the questionnaire included items on education, social status, and the child and family respiratory history. Only one questionnaire was completed per parent or caregiver.

The questionnaire was developed by one author (RMF), and questions were derived from previously used questionnaires [1,10]. Content validity was assessed by a pediatric pulmonologist (TB). A prior pilot study including 20 participants evaluated its acceptance and reproducibility, and results led to minor amendments to its format.

### Sample Size Calculation and Data Analysis

We calculated the sample size for the parent's group based on a rate of 35% parents not identifying "wheezing" through an auditory cue [10]. We estimated a minimum of 400 parent participants would be required to estimate this proportion with a 10% confidence interval (CI) width, assuming 20% of non-responders. There was no sample size calculation for the group of health professionals, and all were approached at each center.

General descriptive analysis of the participants' characteristics was performed. We compared definitions of wheezing in groups and subgroups of participants using the χ^2 ^statistic for categorical variables, and Student t-test for continuous variables. We used bivariate analysis to determine associations between symptoms and *a-priori-*defined participants' characteristics. These variables were entered in multivariate logistic regression models if they were found to be associated at p < 0.2. We report unadjusted and adjusted odds ratio (OR). Participants with incomplete responses were excluded from the analysis of the corresponding parameter, and there was no imputation of missing data. P values < 0.05 were considered statistically significant, and we estimated 95% confidence intervals (CI). All analyses were performed using SPSS for Windows (SPSS Inc, version 15.0).

## Results

### Participants' Characteristics

We approached 900 participants overall. Response rates were over 80% in parents and caregivers and 90% in health care professionals, both comparable between centers. Less than 5% of completed questionnaires were considered invalid.

A total of 721 questionnaires were included in the study, 423 from parents or caregivers and 298 from health care professionals. Professionals were either hospital-based (248/298, 83%) or worked in primary care (50/298, 17%), with 142 physicians and the remainder 156 nurses or physiotherapists. Parents and caregivers were recruited in hospital or community settings (289/423, 68%; and 134/423, 32%, respectively). The former included parents attending general pediatrics (127/289) or pulmonology/allergology visits (66/289). An additional 96 parents were approached in emergency departments or after hospital admission. All participants from community settings were attending well child visits. Most parents and health professionals were recruited in centers from the Lisbon area (74%, 312/423; and 70%, 209/298, respectively).

Table 1 summarizes social, educational and respiratory history data from parents or caregivers and children. Participants were more frequently the child's mother (84%), native Portuguese speakers (96%), with low education levels (82% ≤ 12 years). Seventy-two percent of children were infants or preschoolers. A history of respiratory disease was reported frequently, either in the parent/caregiver (42%) or in children (45%).

**Table 1:** Characteristics of Parents/Caregivers and Children (n = 423)*†

Parents/Caregivers	
Type and age of caregiver	
Parents	402 (95%); 32 years [(28-37)]
Grandparents	13 (3%); 51 years [(48-57)]
Others	8 (2%); 25 years [(18-31)]
≤ 12 years of Education	345 (82%)
First Language Portuguese	406 (96%)
History of Respiratory Disease	178 (42%)

**Children**	

Age	3.5 (0.8-7.8) years
≤ 6 years	277 (68%)
6-10 years	79 (19%)
≥10 years	51 (13%)
Female	187 (46%)
History of Respiratory Disease	183 (45%)

* Age shown in median (P25-75) years; N (%) for the remaining characteristics

†Data available from 407 children

### Recognition of the term "wheezing" by parents and caregivers

Overall, 145 participants (34%; 95% CI 30-39%) reported that they did not recognize the term "wheezing". In bivariate analysis including all reported data, not being familiar with "wheezing" was associated with caregiver's education level ≤12 years (OR 2.6, 95% CI 1.4-5), community setting (OR 2.3, 95% CI 1.4-3.6), no history of respiratory disease in caregivers and children (OR 2.8, 95% CI 1.8-4.3; and OR 6.1, 95% CI 3.8-9.9, respectively), center outside the Lisbon area (OR 2.3, 95% CI 1.5-3.6) and non-native Portuguese language (OR 37, 95% CI 5-280) (all OR values unadjusted). Caregivers who did not recognize the term were younger, as well as their children (t-test, p < 0.001 for both variables). There were no associations with type of caregiver (χ^2^, p = 0.68) or child gender (χ^2^, p = 0.77), and these were excluded from multivariate analysis.

Variables entered in multivariate logistic regression analysis are shown in Table 2, with adjusted ORs. Incomplete responses from 47 participants were excluded for this analysis. Variables associated with not knowing "wheezing" were, in decreasing strength of the association (i.e. decreasing OR): non-native Portuguese language, absence of history of respiratory disease in child, geographical location (South of Portugal vs Lisbon area), lower caregiver educational level, primary care setting, no history of respiratory disease in caregiver, and younger age of caregiver. All variables associated in bivariate analysis were also associated in multivariate models, except for the age of the child.

**Table 2:** Characteristics associated with not recognizing the term "Wheezing": Logistic Regression Analysis (n = 376)*

Independent variable	Recognizes "wheezing"		**Adjusted OR [95% CI]**†	P-value
			
	Yes	No		
First Language				
Non-portuguese	1	14	69.8 [7.9-612.8]	< 0.001
Portuguese	248	113	1	
Caregiver's Education				
< 12 years	191	114	3.3 [1.5-7.4]	0.004
> 12 years	58	13	1	
Setting				
Primary Care Clinic	62	56	3.1 [1.5-6.4]	< 0.001
Hospital	187	71	1	
History of Respiratory Disease (child)				
No	105	105	4.6 [2.5-8.7]	< 0.001
Yes	144	22	1	
History of Respiratory Disease (family)				
No	123	96	2.1 [1.2-3.7]	0.014
Yes	126	31	1	
Geographical Location				
South	199	79	4.5 [2.2-9.1]	< 0.001
Lisbon Area	50	48	1	
Adult's Age (per 10-year increment)			0.4 [0.3-0.7]	< 0.001
Child's Age (per year increment)			1 [0.9-1.1]	0.354

*The models were validated by goodness-of-fit; no interactions were tested. Participants with incomplete answers in any of these parameters were not included.

† OR: Odds-Ratio, CI: Confidence Interval; OR adjusted for all the variables listed

### Meaning of "wheezing" for parents and caregivers

**Figure 1:**
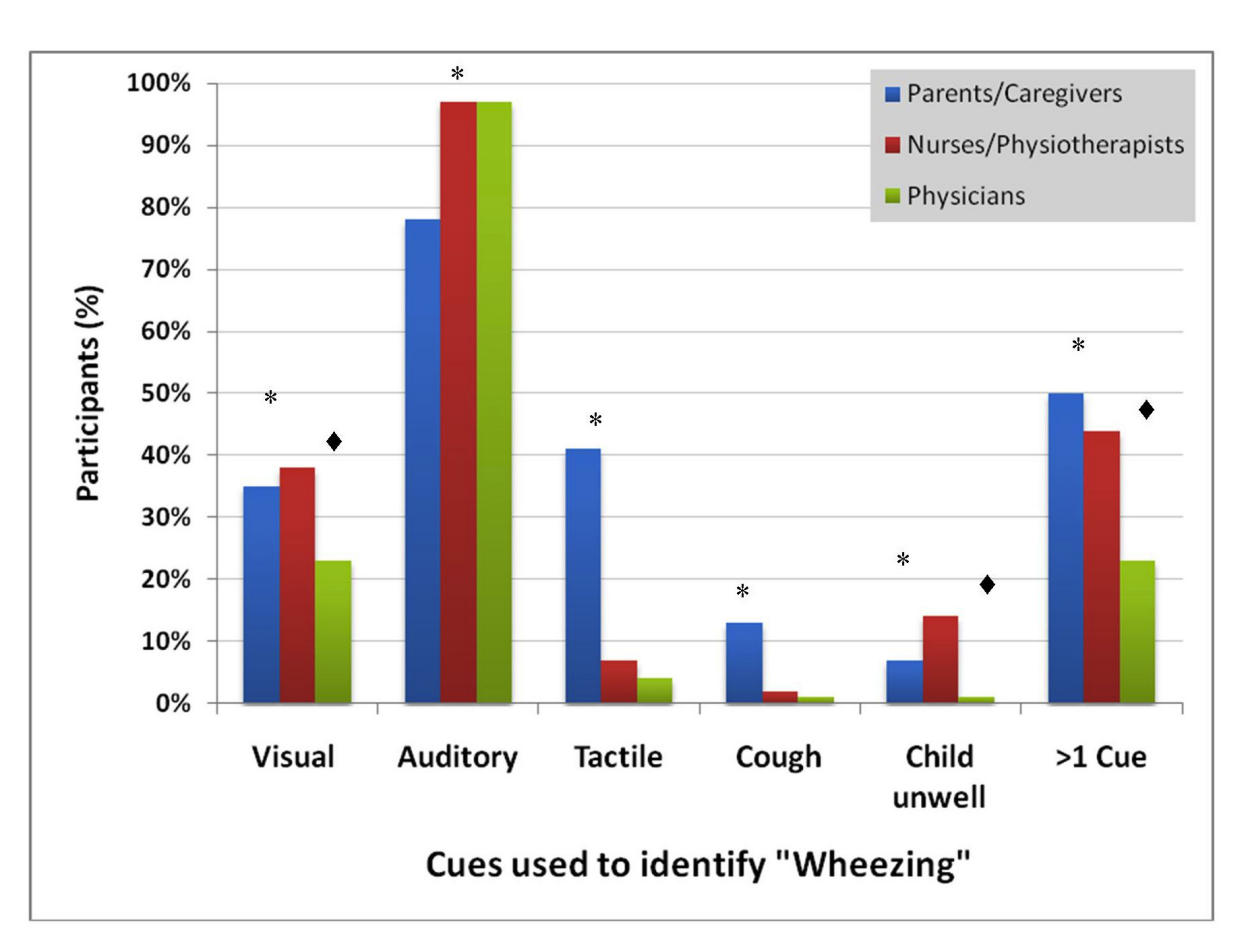
**Cues Used by Parents/Caregivers, Nurses/Physioterapists and Physicians to Identify "Wheezing" (n = 242, n = 155 and n = 142, respectively)**. [black asterisk: statistically significant overall comparisons (χ^2^, p < 0.001); black lozenge: statistically significant comparisons between physicians and nurses/physiotherapists (χ^2^, p < 0.05)].

**Figure 2:**
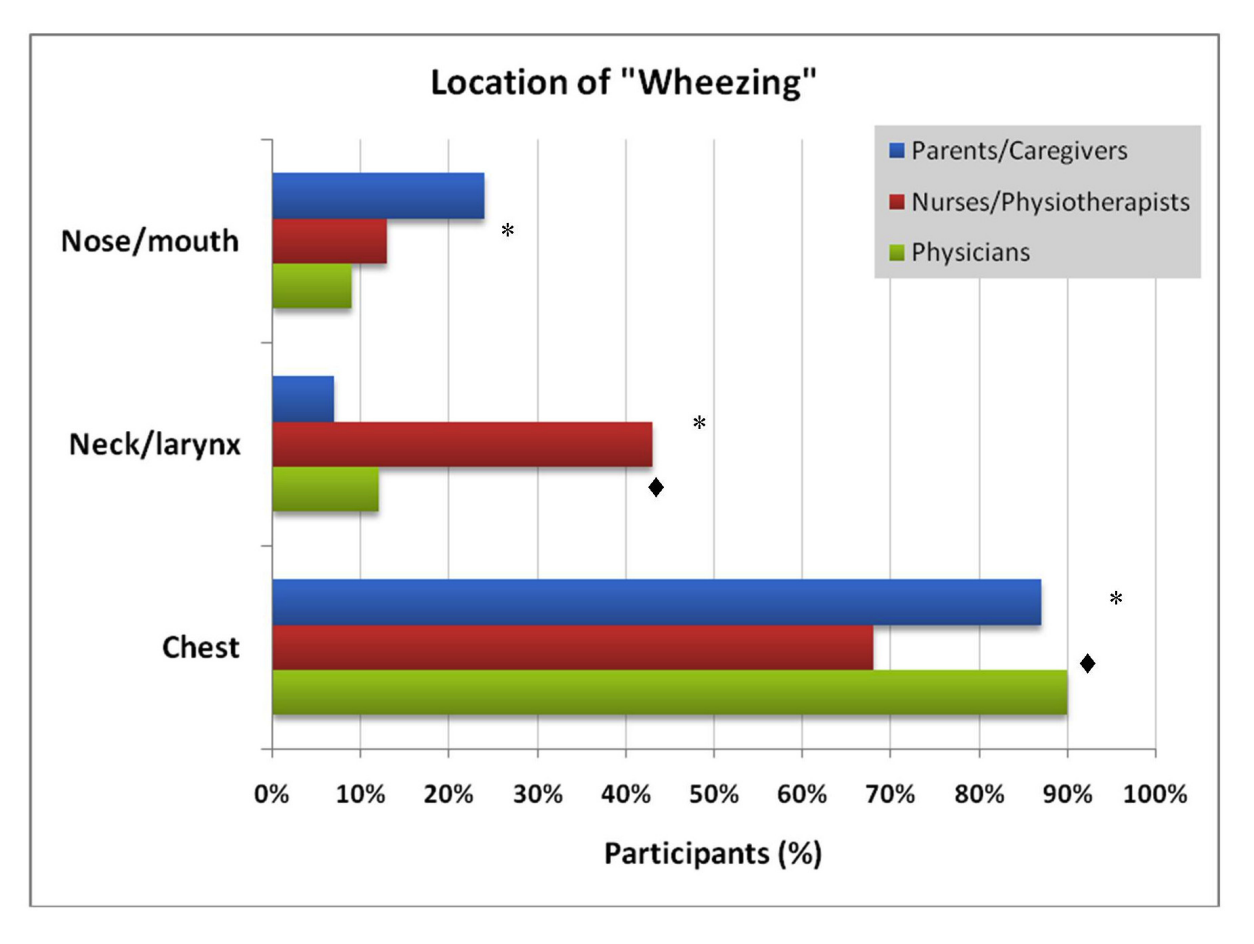
**Location of "Wheezing" for Parents/Caregivers, Nurses/Physioterapists and Physicians (n = 260, n = 155 and n = 140, respectively)**. [black asterisk: statistically significant overall comparisons significant (χ^2^, p < 0.001); black lozenge: statistically significant comparisons between physicians and nurses/physiotherapists (χ^2^, p < 0.05)].

Among 278 parents or caregivers who reported knowing the term "wheezing", valid responses were collected from 242 (87%) on which cues they used to identify it, 260 (94%) on where they located it, and 235 (85%) on both these questions. Results are shown in Figure 1 and 2, respectively. Sixty-nine percent (163/235) participants identified "wheezing" as a sound located in the chest, in agreement with its epidemiological definition. In contrast, 22% (53/242) did not identify it as a sound, and 13% (33/260) did not locate it to the chest. Both components of this definition were absent in 3% (6/235) of participants. Parents or caregivers with lower education more frequently did not identify "wheezing" with auditory cues (unadjusted OR 5.9, 95% CI 1.8-19.8), and no additional statistically significant associations were found with any other parameters.

Parents mentioned using tactile (35%, 100/242) or visual (41%, 84/242) cues to identify "wheezing" (Figure 2). Cough and a sense of being unwell were reported less frequently. More than one cue was used by 50% (120/242) participants. "Wheezing" was located to the nose and mouth by 24% (62/260) parents, and to the neck by 7% (19/260).

As shown in Table 3, respiratory sounds other than "wheezing" were reported as synonyms of "wheezing" by parents and caregivers. Forty-three percent (119/278) mentioned either ruttles- or snoring-like terms, while 20% (56/278) did not identify wheezing-related terms as synonyms. We found associations between the choice of synonyms for "wheezing" and the educational level of caregivers and geographical location, and the child's respiratory history (χ^2^, p < 0.001, for all variables). These associations were preserved in a multivariate model, with a higher risk of reporting synonyms other than "wheezing"-like in parents with ≤12 years of education (adjusted OR 3.7, 95% CI 1.7-7.9), with a child with no respiratory history (adjusted OR 3.1, 95% CI 1.5-6.4), and located outside the Lisbon area (adjusted OR 5.6, 95% CI 1.6-19.7). There was no association between identifying "wheezing" as a sound, and the choice of synonyms (χ^2^, p = 0.85). Parents which mentioned visual and tactile cues more frequently reported ruttles- and dyspnea-related terms, respectively, as synonyms for "wheezing" (χ^2^, p < 0.001 and p = 0.04, respectively).

**Table 3:** "Wheezing" synonyms identified by Parents/Caregivers and Health Care Professionals*†

**Term**‡	Parents /Caregivers (n = 278)	Nurses/ Physiotherapists (n = 155)	Physicians (n = 142)	P
"Wheezing"-related	222 (80%)	152 (98%)	142 (100%)	< 0.001
"Ruttles"-related	97 (35%)	12 (8%)	6 (4%)	< 0.001
"Snoring"-related	22 (8%)	8 (5%)	1 (1%)	0.017
"Dyspnea"-related	19%	20 (13%)	9 (6%)	0.02

*Multiple-choice questions

†Data presented as N (%); χ^2 ^test; other synonyms were chosen in less than 1% of cases in all groups

‡These terms are translations of equivalent respiratory symptoms and sounds in Portuguese; see additional files 1, 2 and 3 for complete questionnaire

The overall reported prevalence of ever "wheezing" in children of participants who reported knowing the term was 51% (139/272). This varied from 36% in well-child community visits, to 56% in hospital settings. Among these participants with valid answers, 20% did not identify "wheezing" as a sound (27/133), 10% (14/136) did not locate it to chest, and 12% (16/139) used synonyms other than "wheezing"-related terms. We did not find any statistically significant association between a history of reported "wheezing" and either identifying wheezing as a sound (χ^2^, p = 0.92) or using other than "wheezing"-related synonyms (χ^2^, p = 0.36).

### Meaning of "wheezing" for health care professionals

Most health care professionals identified "wheezing" as a sound (97%, 288/298) (Figure 1). Twenty-two percent (64/298), however, did not locate it to the chest (Figure 2). This was more frequent with nurses and physiotherapists (32%) than with physicians (10%) (χ^2^, p < 0.001). Additionally, nurses and physiotherapists reported using visual cues (38%) and a sense of being unwell (14%) more often than physicians to identify "wheezing" (χ^2^, p = 0.006 and p < 0.001)) (Figure 1). Use of more than one cue was also more frequent with nurses (44%) than physicians (23%) (χ^2^, p < 0.001). Few health care professionals reported non-"wheezing" related synonyms (Table 3). These were more commonly mentioned by nurses and physiotherapists than physicians (χ^2^, p = 0.006).

## Discussion

Our study shows that parental understanding of "wheezing" differs from most epidemiological definitions, and is distinct from health care professionals' perceptions. These results were obtained using a common Portuguese translation of the term used in a large-scale international questionnaire, and they are consistent with findings from previous studies mostly conducted in English-speaking countries [1,10,12,15,20]. Importantly, a relevant subgroup reported not being familiar with the term "wheezing", and we identified social, clinical and geographical characteristics associated with this outcome. The impact of these different perceptions must be considered when developing and using questionnaire instruments in epidemiological or intervention studies, and can also influence clinical practice.

### Impact of parental perceptions of "wheezing"

In epidemiological studies and clinical practice, "wheezing" is usually defined as a whistling sound located in the chest [9,10,12-14]. We found that 34% parents or caregivers reported not knowing this term, and of those that did recognize it, 31% interpreted it differently. Such differences in conceptual understandings of "wheeze" by parents have been identified in previous studies, both quantitatively and qualitatively [10,12,15,20,21]. Few of these, however, were performed outside the UK [21]. Our study was conducted in a Portuguese population, and we chose a translated term for "wheezing" that is commonly used in both clinical practice and large-scale epidemiological studies. In a recent population-based survey from a respiratory cohort, Michel et al reported a slightly lower proportion of parents not identifying "wheezing" as sound [16]. In their study, however, a definition of "wheezing" was given before the questionnaire, which may explain differences between estimates. Parent's use of other respiratory sounds as synonyms for "wheezing" was also remarkably similar between studies. This suggests that variations of parents' understanding and interpretation of "wheeze" is present cross-culturally, in different settings, and is not exclusively a linguistic issue.

These findings may impact the accuracy with which "wheezing" prevalence rates are estimated through questionnaires alone. Evidence is conflicting when comparing parental assessments of wheezing and other respiratory sounds with different putative clinical "gold-standards". Studies in the acute care setting have shown considerable variation in agreement between clinicians and parents when using the term "wheezing" for the description of acute respiratory symptoms [10,17]. Importantly, one study showing good agreement was conducted in Portuguese- and Spanish-speaking countries [17,22]. Use of video recordings of children presenting with different respiratory sounds improves the accuracy of parental report and confirms that misclassification of sounds by parents is frequent, as there is limited agreement between written and video questionnaires for the term "wheeze" when using the English language [15,23]. Additionally, children with clinically confirmed wheeze in the first years of life later have poorer lung function than those with parent-reported wheezing [24]. Contradictory results may arise from the absence of an accurate "gold-standard" for "wheezing", which also reflects the heterogeneity and different dimensions of asthma and wheezing disorders [25,26]. Michel et al have modeled bias from different degrees of parental misunderstandings of "wheezing" using various hypothetical scenarios, all of which showed considerable impact on epidemiological survey results [16]. Our study adds to these results by showing that a considerable proportion of parents reported not knowing "wheezing". It is reasonable to assume that these parents are at a risk of misclassifying "wheezing" items in questionnaires, although we did not assess the direction or magnitude of bias. This adds complexity to the interpretation of questionnaire results. Further research is needed to assess what is the impact of different perceptions of "wheezing" in the accuracy with which parents recognize and report this symptom, and whether it varies in different settings, languages or cultures.

### Variables associated with parental perceptions of "wheezing"

We identified subgroups of parents which were more frequently unfamiliar with the term "wheezing", based on characteristics which may be classified into three categories: social, clinical and linguistic. Younger and less educated parents or caregivers fit in the first category, and the second includes children attending well-child visits, with no prior history of respiratory disease themselves or their parents. A third category includes non-national first language (Portuguese in this case), and differences in understanding of the term according to geographical location. We hypothesize that the latter are due to region-specific terminologies regarding "wheezing" and other respiratory sounds, since they were independent from setting. Additionally, geographical location was also associated with the pattern of "wheezing" synonyms mentioned by parents. Linguistic considerations have been shown to be important when assessing respiratory symptoms, and there is less congruence across languages for "wheeze" than other terms [19]. Our findings are likely applicable to other languages, including those for which no term for "wheeze" exists, and this evidence strengthens the need for adequate linguistic validation of multicentre and international respiratory questionnaires [27-29]. Overall, the subgroups we identified may be more prone to have biased estimates, and careful interpretation of survey results is warranted. Additional guidance in questionnaires may facilitate the understanding of the term in these populations.

The educational level of parents was the only variable associated with both being unfamiliar with "wheezing" and describing it inadequately, and no other associations were found with the latter. Accuracy of "wheezing" description has been shown to vary based on ethnic, cultural, clinical and linguistic parameters, as well as with the child's respiratory history, i.e. frequency and severity of previous "wheezing" [16]. The fact that we excluded participants which reported not knowing wheezing may have limited the power to investigate these associations. Most predictors identified by Michel et al were consistent with the parameters we found associated with not knowing "wheezing", which reflects an overlap between not knowing the term and defining it inadequately [16]. Educational levels of parents were associated with all study outcomes, in line with findings from qualitative studies highlighting the relevance of social, cultural and linguist backgrounds in parental understanding of respiratory symptoms [20,21,30].

### Cues used by parents and health professionals to identify "wheezing"

Our findings show parents use multiple cues to identify "wheezing", which is consistent with previous results [10]. Visual and tactile cues were often reported, and their association with the use of non-"wheezing" synonyms suggests that parents may confound different respiratory sounds. We also found differences between physicians and nurses/physiotherapists regarding the definition and location of "wheezing", which may have an impact in clinical practice. Of interest is the fact that some health professionals also often used visual cues to define "wheezing". This supports the variability in assessing this symptom, and highlights the difficulty in capturing the concept of "wheezing" with a single definition. The relevance of these cues and their validity when assessing wheezing and asthma in epidemiological studies should be considered for future questionnaires, with more precise and explicit symptom definitions.

### Limitations

This study did not use a large population-based approach, but relied on convenience sampling. However, we sought a priori to recruit participants from different social, clinical and geographical backgrounds, in different clinical settings. There was a high prevalence of wheezing in children of participants, possibly due to the large hospital-based population. We could expect, however, that this would overestimate adequate knowledge of "wheezing". We studied the understanding of respiratory symptoms, but did not compare them to any objective finding. Furthermore, our questionnaire used closed directed questions, which may have missed qualitative aspects of parental or caregiver perception. Our purpose was to mimic approaches susceptible of being used in larger-scale questionnaires, as well as to perform quantitative analysis. Our results were mostly based on parents of younger aged children. Other studies have assessed the accuracy with which children and adolescent perceive and self-report respiratory symptoms [31].

## Conclusions

Parental definitions of "wheezing" differ from epidemiological definitions. Different meanings for "wheezing" are recognized in Portuguese, and may be influenced by factors such as the caregiver's educational background, the child and parent's respiratory history, and geographical terminology. These findings are likely applicable to other non-English languages, and suggest the need for more accurate questionnaires and additional objective measurement instruments to study the epidemiology of wheezing disorders.

## Competing interests

The authors declare that they have no competing interests.

## Authors' contributions

All authors read and approved the final manuscript. *Study concept and design*: RMF, TB; *acquisition of data*: RMF, BR, CC, SM, AS, JF, RR, CB; *analysis and interpretation of data*: RMF, TB; *drafting of the manuscript*: RMF; *critical revision of the manuscript for important intellectual content*: TB; *statistical analysis*: RMF; *funding*: RMF, TB; *study supervision*: RMF, TB

## Pre-publication history

The pre-publication history for this paper can be accessed here:

http://www.biomedcentral.com/1471-2431/11/112/prepub

## Supplementary Material

Additional file 1 **Questionnaire for nurses and physiotherapists**. Questionnaire used for nurses and physiotherapists, in Portuguese. Click here for file

Additional file 2 **Questionnaire for physicians**. Questionnaire used for physicians, in Portuguese. Click here for file

Additional file 3 **Questionnaire for parents**. Questionnaire used for parents, in Portuguese. Click here for file
