# Supplementary material for: The multiple meanings of "wheezing": a questionnaire survey in Portuguese for parents and health professionals
Source: BMC Pediatr. 2011 Dec 12;11:112. doi: 10.1186/1471-2431-11-112 (PMC3266641; doi:10.1186/1471-2431-11-112)
Supplement: Additional file 2 — Questionnaire for physicians. Questionnaire used for physicians, in Portuguese. [file 1471-2431-11-112-S2.DOC]

**SINTOMAS RESPIRATÓRIOS PEDIÁTRICOS**

**Inquérito**

Pretendemos com este inquérito conhecer melhor a **perspectiva médica e parental** sobre os sintomas respiratórios pediátricos,

e contamos com a sua participação.

O questionário é **anónimo**, **não é um teste**, e pode ser preenchido em **minutos**.

Basta **assinalar com uma cruz** as respostas que melhor se aplicam,

ou **preencher com números ou em poucas palavras** no espaço indicado.

Quando o completar, **guarde-o e entregue-o** ao membro da equipa de trabalho presente na sua Unidade.

A equipa da Clínica Universitária de Pediatria

da Faculdade de Medicina de Lisboa **agradece a sua participação**.

1. Qual o seu grau de diferenciação na carreira médica? (assinale com uma cruz a resposta adequada)

🞎 Estudante/Estagiário/Internato Comum

🞎 Interno de Pediatria

🞎 Pediatra

🞎 Interno/Especialista de Medicina Geral e Familiar

🞎 Outra especialidade: _____________________________________________________

1. Qual é a sua **língua materna**?

🞎 Português

🞎 Outra. Especifique qual: ________________________________________________

1. O que entende por ***pieira***? (preencha em poucas palavras)

____________________________________________________________________________________________________________________________________________________________________

1. Reconhece que uma criança tem ***pieira***…? (escolha uma ou mais respostas que se aplicam)?

🞎 identificando dificuldade respiratória no exame objectivo

🞎 por um ruído respiratório audível (com e/ou sem estetoscópio)

🞎 pela palpação torácica

🞎 pela presença de tosse

🞎 pela sensação da criança não estar bem

1. São sinónimos de ***pieira***? (escolha uma ou mais respostas que melhor se aplicam)

🞎 “gatinhos no peito”/”chiadeira”

🞎 expectoração

🞎 farfalheira

🞎 roncopatia

🞎 dispneia

🞎 sibilância

🞎 Outros: _____________________________________________________________

🞎 Nenhum destes

1. Ouve ou sente a ***pieira***? (escolha uma ou mais respostas que melhor se aplicam)

🞎 a nível torácico

🞎 a nível da laringe/pescoço

🞎 a nível nasal/oral

**OBRIGADO PELA SUA PARTICIPAÇÃO**
