# Supplementary material for: The multiple meanings of "wheezing": a questionnaire survey in Portuguese for parents and health professionals
Source: BMC Pediatr. 2011 Dec 12;11:112. doi: 10.1186/1471-2431-11-112 (PMC3266641; doi:10.1186/1471-2431-11-112)
Supplement: Additional file 3 — Questionnaire for parents. Questionnaire used for parents, in Portuguese. [file 1471-2431-11-112-S3.DOC]

**PERSPECTIVA PARENTAL**

**SOBRE OS SINTOMAS RESPIRATÓRIOS EM CRIANÇAS**

**Inquérito**

Pode **ajudar-nos** a conhecer melhor os sintomas respiratórios das crianças preenchendo este inquérito.

É **anónimo**, **não é um teste**, e pode ser preenchido em **poucos minutos**.

Basta **assinalar com uma cruz** as respostas que melhor se aplicam,

ou **preencher com números ou em poucas palavras** no espaço indicado.

Quando o completar,

**guarde-o e entregue-o** depois ao médico da consulta.

A equipa da Clínica Universitária de Pediatria

da Faculdade de Medicina de Lisboa **agradece a sua participação**.

1. Indique o local onde está a preencher este inquérito (escolha com uma cruz uma resposta)

🞎 Consulta de Alergologia ou Pneumologia pediátrica

🞎 Consulta de Pediatria Geral ou de Saúde Infantil

🞎 Serviço de Urgência Pediátrica

🞎 Internamento de Pediatria

🞎 Outro: _____________________________________________

1. Qual é a **idade da criança**? (complete com dígitos) 🞎🞎 anos 🞎🞎 meses
2. Qual é a **sua idade**? 🞎 🞎 anos
3. Assinale o **género** da criança:

🞎 Menina

🞎 Menino

1. Qual é o **seu grau de escolaridade**?

🞎 Até ao 9º ano

🞎 10º ao 12º ano

🞎 Licenciatura, Mestrado ou Doutoramento

1. Qual é a sua **língua materna**?

🞎 Português

🞎 Outra. Especifique qual: ________________________________________________

1. Qual é a sua **relação** com a criança que hoje acompanha?

🞎 Mãe

🞎 Pai

🞎 Avô ou avós

🞎 Outro familiar ou amigo

1. Vive na **mesma casa** que a criança?

🞎 Sim

🞎 Não

1. A **criança** que acompanha já teve ou tem **problemas respiratórios**?

🞎 Sim

🞎 Não

1. Alguém na **família próxima** da criança (incluindo você) tem **problemas respiratórios**?

🞎 Sim

🞎 Não

1. Já ouviu falar na palavra ***pieira***?

🞎 Sim

🞎 Não

**☞ Se respondeU “SIM” 🡺 CONTINUE NA PERGUNTA 12.**

**SE RESPONDEU SIM À PERGUNTA 11**

1. A criança que acompanha já teve ou tem ***pieira***?

🞎 Sim (Se Sim, há quanto tempo teve ou tem ***pieira***? 🞎 🞎 anos 🞎 🞎 meses)

🞎 Não

1. O que entende por ***pieira***? (preencha em poucas palavras)

____________________________________________________________________________________________________________________________________________________________________

1. Reconhece que a sua criança tem ***pieira***…? (escolha uma ou mais respostas)?

🞎 porque a vê com dificuldade em respirar/falta de ar?

🞎 pelo som/ruído que ouve

🞎 pelo que sente no peito dela

🞎 porque ela tosse

🞎 porque percebe que ela não está bem

1. ***Pieira*** é igual a…? (escolha uma ou mais respostas)

🞎 “gatinhos no peito”/”chiadeira”

🞎 expectoração

🞎 farfalheira

🞎 ressonar

🞎 falta de ar

🞎 sibilância

🞎 Outros: _____________________________________________________________

🞎 Nenhum destes

1. Ouve ou sente a ***pieira***? (escolha uma ou mais respostas)

🞎 no peito

🞎 no pescoço

🞎 no nariz/boca

**OBRIGADO PELA SUA PARTICIPAÇÃO**
